# Glucocorticoid Effects on Proteoglycans and Glycosaminoglycans

**DOI:** 10.3390/ijms232415678

**Published:** 2022-12-10

**Authors:** Anastasia V. Strokotova, Elvira V. Grigorieva

**Affiliations:** Laboratory of Glycobiology, Institute of Molecular Biology and Biophysics, Federal Research Center of Fundamental and Translational Medicine (FRC FTM), 630117 Novosibirsk, Russia

**Keywords:** extracellular matrix, tumor microenvironment, glucocorticoid, proteoglycan, glycosaminoglycan, heparan sulfate, chondroitin sulfate, brain

## Abstract

Glucocorticoids are steroid hormones that play diverse roles in numerous normal and pathological processes. They are actively used to treat a wide variety of diseases, including neurodegenerative and inflammatory diseases, cancers, and COVID-19, among others. However, the long-term use of glucocorticoids is associated with numerous side effects. Molecular mechanisms of these negative side effects are not completely understood. Recently, arguments have been made that one such mechanisms may be related to the influence of glucocorticoids on O-glycosylated components of the cell surface and extracellular matrix, in particular on proteoglycans and glycosaminoglycans. The potential toxic effects of glucocorticoids on these glycosylated macromolecules are particularly meaningful for brain physiology because proteoglycans/glycosaminoglycans are the main extracellular components of brain tissue. Here, we aim to review the known effects of glucocorticoids on proteoglycan expression and glycosaminoglycan content in different tissues, with a specific focus on the brain.

## 1. Introduction

Glucocorticoids (GCs) are steroid hormones that regulate multiple physiological functions in all mammalian tissues, including development, immunomodulation, maintenance of circadian rhythm, and the response to stress [1,2]; connect external environmental stress signals with the function of many cell types, which produces profound changes in immune cells, including macrophages [3]; maintain energy homeostasis in multiple tissues, including those in the liver and skeletal muscle, as well as white and brown adipose tissues [4]; maintain tissue homeostasis; and regulate metabolism, cardiovascular and neural function, reproduction, and immune activity [5]. GCs are used to treat a variety of pathologies, such as inflammatory diseases [6], multiple sclerosis [7], systemic lupus erythematosus [8], lupus nephritis [9], Duchenne muscular dystrophy [10], rheumatoid arthritis [11,12], multiple myeloma [13], pediatric acute lymphoblastic leukemia [14], and respiratory pathologies [15], or as a part of complex therapy to treat SARS-CoV-2 infection [16,17] and various cancers including glioblastoma multiforme (GBM) [18,19]. The above demonstrates the usefulness and importance of GCs in the treatment of various pathologies, which is described in detail in numerous reviews devoted to various aspects of the therapeutic use of GCs and the molecular mechanisms of their action.

However, long-term use of GCs is associated with delayed negative side effects at the molecular, cellular, and clinical level [20,21,22], such as undesirable metabolic complications, including the development of type 2 diabetes mellitus [23] and insulin resistance [24], accelerated generalized bone loss and increased vertebral and non-vertebral fracture risk [25,26], and predisposition to cardiovascular diseases [27]. This has become especially important recently due to the active use of GCs during the COVID-19 pandemic and seems to be realized through numerous molecular pathways and mechanisms, including effects of GCs on various cell types and the extracellular matrix (ECM) of any tissue or organ. Whereas the effects of GCs on physiology and functional activity of different cells are being actively studied; however, its effect on the ECM and especially glycosylated components, such as proteoglycans (PGs) and glycosaminoglycans (GAGs), remains very poorly understood. Furthermore, these complex protein–carbohydrate macromolecules play a very important role in the structure of the ECM of all body tissues [28] and largely determine their functional capabilities [29]. PGs are closely involved in the process of malignant transformation, whereby significant changes occur in their composition, content, and structure [30,31,32].

The aim of this review is to provide an overview of the data on the effects of GCs on glycosylated macromolecules, with a special focus on brain PGs and GAGs as the main components of the brain extracellular matrix and tumor microenvironment.

## 2. Overview of the Literature Data

**Figure 1:**
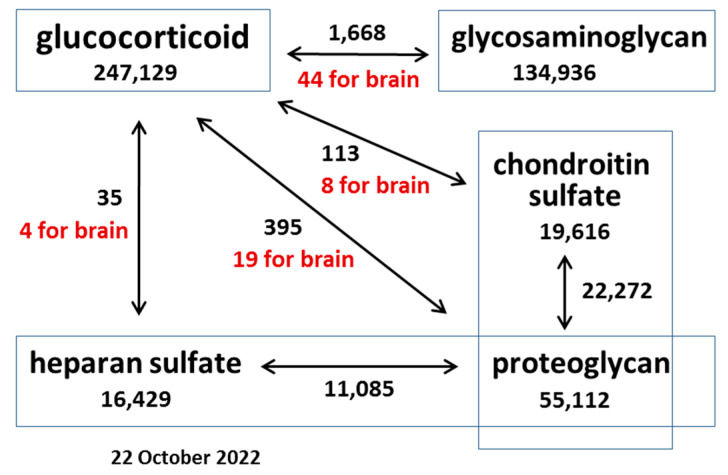
Scheme representing the relative number of publications related to the effects of GCs on O-glycosylated molecules different cells and tissues as of 22 October 2022. Keywords are in rectangles, and the number below a keyword represents the number of publications retrieved from the PubMed database. Arrows indicate a combination of keywords, and the number next to an arrow indicates the number of publications for each combination of keywords (of which a certain number includes the additional keyword “brain” (highlighted in red)).

Because GCs are among the most used drugs in the world, their effects on various organs and tissues have been actively studied for many years. A search using the PubMed query “glucocorticoid” returned 247,129 results as of 22 October 2022, of which 33,278 are literature reviews, including 1100 for year 2022 (Figure 1). 

The study of PGs and GAGs is also of considerable interest, with a large number of works published to date—55,112 articles for “proteoglycan”, 22,272 for “chondroitin sulfate proteoglycan”, and 11,085 for “heparan sulfate proteoglycan” (PG, CSPG, and HSPG, respectively).

However, the use of these terms in combinations returned significantly fewer articles than for each of them separately. Whereas 247,129 articles are dedicated to the study of GCs, the influence of GCs on PGs returns only 395 articles, only 19 of which are relevant to the study of these processes in the brain. With respect to specific PG types (HSPG and CSPG), the search yields an order of magnitude fewer articles (35 and 113, respectively).

A particularly small number of publications has been published in the field of research on the effects of GCs on PGs and GAGs in the brain, representing an obvious gap in the literature.

An overview of studies performed in this area of research convincingly shows that despite the enormous interest and progress in the study of the functional role and the molecular mechanisms of action of glucocorticoids, their effects on glycosylated molecules (such as PGs and GAGs), especially in the brain, remain underinvestigated.

## 3. Effects of GCs on PG Expression and GAG Content in Normal Tissues and Cells

The effect of GCs on PG expression and GAG content has been shown for various normal cells and tissues, especially in tissues with high GAG content, such as cartilage/chondrocytes and lung tissue/fibroblasts. Various synthetic GCs are used to study the effects of GCs, including dexamethasone (DXM), betamethasone, prednisolone, budesonide, triamcinolone, and hydrocortisone, among others. 

### 3.1. Cartilage and Chondrocytes

GAGs comprise a significant part of cartilage tissue and play an important role in its organization and functioning. The effects of GCs on the GAG content in cartilage from knee joints were studied in Göttingen miniature pigs. Short-term prednisolone treatment at a dose of 1 mg/kg/day for 2 months and 0.5 mg/kg/day for 6 months did not affect GAG content in the cartilage, whereas long-term prednisolone treatment at a dose of 1 mg/kg/day for 2 months and then 0.5 mg/kg BW/day for 13 months resulted in a decrease in GAG content by 14% (*p* < 0.04) [33]. In a study involving multiple injections of betamethasone into the knee of Californian rabbits, only 6–8 injections with a 1-week interval significantly decreased total PG content in the articular cartilage from tibial plateaus, whereas 1–5 injections do not change the PC content [34]. Four repeated methylprednisolone acetate (MPA) injections (at 0, 5, 10, and 15 weeks of the experiment) with a single dose of 80 mg/mL led to significant prolonged (20 weeks) loss of PG content (*p* = 0.008) in female Suffolk cross sheep in a controlled partial anterior cruciate ligament (*p*-ACL) injury model [35]. These results show an inhibitory effect of GCs towards GAG/PG content in cartilage; however, when used in combination with other drugs/cartilage destructors, GCs demonstrate protective properties with respect to GAG/PG content. Therefore, in the animal model of combined damage of cartilage (mechanical injury, tumor necrosis factor alpha (TNFα) and interleukin-6 (IL-6)/soluble IL-6 receptor (sIL-6R)), DXM dose-dependently (at 100 nM and higher) inhibits both TNFα-induced GAG loss and a reduction in biosynthesis in bovine and human cartilage explants to control levels. Moreover, DXM pretreatment or post-treatment of bovine explants inhibits GAG loss and increases PG synthesis in cartilage explants exposed to TNFα, preventing cartilage degradation [36]. GCs also restored the decreased content of total GAGs in rats temporomandibular joints in an in vivo model of acute temporomandibular joint osteoarthritis [37]. A similar result was shown for rat knee joints, where DXM administration at a dose of 1 mg/kg immediately prior to TNFα injection prevented TNFα-induced degradation of CSPG aggrecan and the release of aggrecanase-generated aggrecan fragments from the articular cartilage into the synovial fluid [38]. DXM treatment at 2.5 mg/kg for five cycles for 5 consecutive days with breaks of 16 days also attenuated TMZ-induced reorganization of knee joint cartilage structure (increased content of total GAGs and their partial relocalization from chondrocytes into the tissue matrix, as well as a decrease in sulfated GAG content) in elderly Wistar rats [39]. Triamcinolone acetonide (TA) at doses of 1.25, 2.5, and 5 mg/mL increased PG loss in canine cartilage explants [40].

In addition to studying the content of total PGs and GAGs in cartilage tissue, a significant number of studies have been conducted on primary cultures of chondrocytes obtained from cartilage. A low dose of TA (0.11 mg/mL) induced downregulation of aggrecan in primary canine normal chondrocytes, whereas it upregulated aggrecan expression in canine osteoarthritic chondrocytes [40]. In chondrocyte primary cultures from articular cartilage from metacarpophalangeal and metatarsophalangeal joints of 1–10-year-old horses, short-term use of MPA at a dose of 0.05 and 0.5 mg/mL decreased production of total PGs by 37% and 75%, respectively. Glucosamine at doses of 0.5 mg/mL and 10 μg/mL significantly increased PGs production, demonstrating a protective effect against MPA-induced PG inhibition. These data suggest that glucosamine may be useful as an adjunct treatment when an intra-articular injection of a corticosteroid is indicated [41]. Short DXM treatment (10^−6^, 10^−7^, or 10^−8^ M for 24 h) of chondrocytes isolated from mouse knee joints induced a two- to threefold dose-dependent decrease in content of total PGs in the cell culture in vitro [42]. Treatment of human chondrocytes from knee joints with 50–150 μM DXM for 1–7 days resulted in a time-dependent and dose-dependent decrease in aggrecan content both at the mRNA and protein levels, likely through induced p38 inactivation [43]. Daily treatment of pregnant spiny mice with 125 μg/kg DXM per day (beginning on gestation day 20 until parturition) decreased the amount of total PGs in articular and growth cartilages of the offspring [44]. Overexposure of pregnant C57BL/6 mice to different doses of DXM (0, 0.2, 0.8, and 1.2 mg/kg/day) once a day from day 12 to 17 of gestation significantly reduced gene and protein expression of aggrecan in offspring and suppressed articular cartilage development in a course-, dose-, and stage-dependent manner [45].

Despite the fact that most of the mentioned works talk about the negative effect of GCs on the PG content, which is related mainly to a decrease in total PG/GAG content and aggrecan expression, some works show opposite effects. For example, it was shown that DXM at a dose of 1 µM led to an increase in GAG production in primary bovine articular chondrocytes on day 21 (*p* < 0.001) and chondrogenically differentiated mesenchymal stem cells in a 3D alginate cell culture model on day 14 (*p* < 0.01) and from day 21 to 35 (*p* < 0.0001) [46]. Treatment of equine articular chondrocyte primary cultures from foals, as well as 7-year-old and adult horses, with low doses of GCs (DXM, triamcinolone, and prednisolone) at doses of 10^−10^ to 10^−4^ M for 24 h did not affect *Biglycan* and *Decorin* expression [47]. Long-term treatment with 100 nM DXM for 21 days prevented up to 67% of loss of sulfated GAGs from injured and cytokine-treated human osteochondral explants to the cultured medium, with donor-specific differences in the sulfated GAG attenuation effect in an ex vivo human osteochondral model of post-traumatic osteoarthritis [48]. 

In general, the question of the effects of GCs on PGs in cartilage and chondrocytes has been studied in more detail than for other tissues, as demonstrated by the publications presented above and presented in generalized form in Table 1.

Despite the relatively large number of articles presented deep analysis and the integration of the known results into whole picture is difficult, as many parameters are involved in these studies simultaneously (such as specific GC, dose, treatment regimen, time of the final analysis, animal/tissue/cell type, studied parameters, etc.). As a result, the available publications have almost unique combinations of these parameters and do not form any groups that allow us to draw generalizing conclusions. The literature comprises unique studies that are difficult to combine into one holistic picture.

Nevertheless, it is possible to observe a trend that GCs decrease PG/GAG content in cartilage, and aggrecan was identified as a main GC-responsive PG in this tissue.

### 3.2. Lungs and Fibroblasts

Glucocorticoid signaling via the glucocorticoid receptor (GR) is essential for lung maturation in mammals. One of the downstream GC targets in conditional mesenchymal GR-deficient mouse lungs is CSPG *Versican* (*Vcan*), an important extracellular matrix component and cell proliferation regulator. Coordinately acting GC steroids regulate the repression of *Vcan* gene expression and *Vcan* proteins to contribute to the normal development of the fetal respiratory system in mammals [72]. Both GR and its transcriptional target, *Versican*, were shown to be critically important for the regulation of respiratory maturation and, ultimately, survival at birth of experimental mice [73]. To study whether GC-induced reductions in perialveolar tissue volumes are associated with *Versican* expression levels or sulfation patterns of its chondroitin sulfate (CS) side chains, fetal sheep were infused with cortisol at 1.5–4 mg/day for a period of 9 days or treated with GC betamethasone at 11.4 mg for 24 h and 36 h. Betamethasone decreased *Versican*, chondrotin-6-sulfate (C-6-S), and chondroitin-4-sulfate (C-4-S) levels in the fetal sheep lungs compared with saline-exposed fetuses, whereas cortisol decreased C-6-S but did not significantly alter the level of *Versican* or C-4-S [50].

The indirect influence of GCs on PG expression was studied in an experimental system of activation of PG production in human lung fibroblasts by serum. An increase in serum concentration up to 10% (compared to the basic 0.4%) resulted in a 1.5-fold increase in total PG production and a 2.5- to 5-fold increase in PGs *Decorin*, *Biglycan*, perlecan, and *Versican* expression. Administration of GC budesonide at a dose of 10^−8^ M for 24 h reduced the serum-induced total PG increase by 44% (compared to the 10% serum-induced level) and *Decorin* and *Versican* levels (up to 39% and 40% of basal expression, respectively) [51]. Short-term treatment (18 h) of mouse fibroblasts with GCs DXM, methylprednisolone, budesonide, or fluticasone increased expression of accessory type III TGF-β receptor Tgfbr3, also called *Betaglycan*, by 1.9-fold. Intraperitoneal administration of 20 nM DXM for 24 h to C57Bl/6J mice resulted in an increase in *Betaglycan* content in mRNA and protein levels exclusively in the lungs but not in the extrapulmonary organs [49].

Overall, in the lung tissue, GCs affect the expression of *Versican* and sulfation of its CS chains, along with the activation of the expression of accessory type III TGF-β receptor *Betaglycan*.

### 3.3. Dermal Fibroblasts

The effect of GCs on the expression of PGs and GAG content in fibroblasts from the skin has been studied much less.

According to Kahari et al., in cultured human skin fibroblasts, 1 µM DXM increased *Decorin* production both in mRNA (up to 2.3-fold) and protein (up to 4.8-fold) levels but had no effect on *Biglycan* production or the length of CS/DS side chains attached to *Decorin* and *Biglycan* core proteins. Moreover, DXM attenuated effects of TGFβ on these PGs by preventing the TGFβ-induced downregulation of *Decorin* and upregulation of *Biglycan* in dermal fibroblasts. These results demonstrate that the GC effects on dermal connective tissue are partially mediated by altered expression of *Decorin* and *Biglycan*, both of which, in turn, regulate the activity of TGFβ, the most potent stimulator of connective tissue deposition [52].

Treatment of human skin fibroblasts with GCs fluocinolone acetonide and budesonide, even at low concentrations (10^−11^–10^−5^ M) strongly reduces the accumulation of hyaluronic acid (HA) and the content of sulfated GAGs in the culture medium, at the cell surface, and in the cells, whereas hydrocortisone has considerably less effect. The most drastic changes were shown for dermatan sulfate (DS), where GC treatment both decreased DS content in all three compartments and changed the DS chain structure through a decrease in glucuronic acid (GlcUA) and an increase in iduronic acid (IdoUA) residues. HA and heparan sulfate (HS) were specifically decreased in the culture medium and retained on the cell surface. Overall, GC treatment not only decreases the GAG content in human fibroblasts but also changes their structure and distribution, probably contributing to the development of skin atrophy, which is often observed after long-term GC treatment [54]. The observed loss of HA in skin fibroblasts could be related to the expression and/or enzymatic activity of their biosynthetic enzymes. It was shown that incubation of cells for 24 h in the presence of 1 µM DXM induces rapid (1–2 h) and sustained, near-total (97 ± 98%) suppression of human hyaluronan synthase 2 (HAS2) (responsible for HA biosynthesis) in cultured human dermal fibroblasts and the MG-63 osteoblast-like osteosarcoma cell line through substantial decreases in both gene transcription and message stability [53].

In summary, the presented data identify *Decorin* and *Biglycan* as main GC-responsive genes in dermal fibroblasts, as well as GAG chains of HS, CS, and HA.

### 3.4. Osteoblasts and Bone Marrow Stromal Cells

GCs have marked effects on ECM protein synthesis in bone, and PGs are important components of the bone matrix, taking part in the differentiation and biological activities of osteoblasts. DXM treatment at doses of 10^−10^–10^−7^ M for 7 days resulted in a fast and dramatic increase in *Decorin* and a decrease in *Biglycan* expression and protein content in both the conditioned medium and the cell layer of human osteoblasts (HOB) and adult human bone marrow stromal cell (BMSC) cultures in a dose- and time-dependent manner [55]. 

In osteoblast-like and preosteoblast-like cell lines (murine MC3T3-E1 and rat RCT1, respectively), DXM treatment (10^−10^–10^−7^ M) rapidly (9–48 h) and dose-dependently increased expression of HS/CSPG *Betaglycan* (type III TGFβ receptor) and its binding with TGFb1 [74]. In immature, non-transformed mouse bone-marrow-derived mast cells (mBMMC), DXM dose- and time-dependently stimulated (threefold) the expression of CS/DS/HS proteoglycan serglycin [56]. Furthermore, maternal DXM (0.2 mg/kg once a day from gestational day 9 to 20) decreased total PG content in the growth plate of fetal rats. Lengths of fetal femurs and growth plates were both 19.19% shorter in the fetuses, affecting the development of growth plates and fetal long bones [57].

The effects of DXM on ECM production in a tissue engineering culture system were found to be dependent on cell type and their microenvironment. It was shown that 100 nM DXM stimulated PG biosynthesis and retention in agarose hydrogels seeded with young bovine bone marrow stromal cells (BMSCs) but decreased PG accumulation in peptide scaffolds. DXM-treated adult human BMSCs showed minimal matrix accumulation in agarose but accumulated ~50% as many PGs and collagen as young bovine BMSCs in peptide hydrogels [58]. Comparative analysis of the three hematopoietic-supportive bone marrow stromal cell lines D2XRII, Bl6, and 14F1 revealed a significant variability of the cells in terms of PG composition and content. All cultures synthesized three species of CS/DS PGs, with dermatan sulfate 1 (DS1) and dermatan sulfate 2 (DS2) present mainly in the culture media and dermatan sulfate 3 (DS3) present mainly in the cell layers. CSPGs/DSPGs are the major PGs in all cultures; hydrocortisone-free cultures also synthesize two different HSPGs denoted as heparan sulfate 1 (HS1) and heparan sulfate 2 (HS2). Hydrocortisone treatment at a dose of 1 μM abolishes HSPG synthesis in all three cell lines and alters the pattern of CS/DSPGs in the culture media, increasing the quantity of DS1 and DS3 and reducing the quantity of DS2 [59]. 

In general, the few existing studies that are cited here are relatively old and describe the effect of GCs on completely different PGs and GAGs. The lack of up-to-date publications reflects a noticeable gap in this area of research.

### 3.5. Other Tissues and Cells

PGs are integral components of the mesangial matrix and glomerular permeability barrier and play a significant role in the pathogenesis of renal disease. Steroid hormones (used as first-choice therapy for the treatment of glomerular diseases) resulted in a dose- and time-dependent 50% decrease in the total CS/DS PGs synthesis and secretion in rat mesangial cells (RMCs) and human mesangial cells (HMCs), as well as *Decorin* core protein expression, whereas *Biglycan* expression was increased after the DXM treatment. A similar trend was found in glomeruli isolated from rats treated in vivo [67]. Administration of methylprednisolone at the onset of murine lupus nephritis (at about 5 months of age) decreased the expression, distribution, and intensity of HSPG staining in the renal cortex of New Zealand Black/White F1 mice by 50%, *p* < 0.05 [70].

A similar tendency of suppression of PG biosynthesis upon GC administration was shown for primary cultures of human tenocytes from explants of healthy human patellar tendon. Treatment of the cells with 1 μM triamcinolone or 1 μM DXM reduced total PG content of control cultures by 80% (*p* = 0.007) and 72% (*p* = 0.01), respectively, potentially affecting the viscoelastic properties of tendon and increasing the risk of spontaneous rupture [69].

Long-term ex vivo administration of DXM at a dose of 0.55 μM to human eyes in perfusion organ culture resulted in a delayed twofold increase in total GAG (CS, DS, HS, and HA) content (*p* = 0.03) in human trabecular meshwork 14–21 after days treatment, whereas such a change was not apparent in eyes treated for 7 days [66].

In hepatic stellate cells (HSCs), GCs specifically increased the expression level of *Betaglycan* (TGFβ receptor type III) in a time- and dose-dependent manner but not TGFβ receptor types I and II. The effect is dependent upon the nature of the stimulating hormone: DXM administration at doses of 200 or 400 ng/mL of medium for 4–8 h resulted in a 7.8–13.1-fold increase in the *Betaglycan* mRNA level, whereas hydrocortisone administration at a dose of 200 ng/mL or aldosterone administration at a dose of 500 ng/mL increased the *Betaglycan* mRNA level only by 3.5-fold and 2.9-fold, respectively [68].

Recently, several interesting studies have been conducted on the effect of GCs on PG expression and GAG content in human tissues [65,71]. A single preoperative dose of 2 mg/kg methylprednisolone to patients undergoing gastrointestinal surgery reduced the expression level of HSPG syndecan-1 and HS content in endothelial glycocalyx at 1 and 3 days after surgery [71]. In another clinical trial, Yanase et al. demonstrated that a single 16 mg preoperative administration of DXM to patients undergoing colorectal, pancreas, or liver surgery did not affect plasma syndecan-1 levels on postoperative day 1 but significantly (four to fivefold) decreased HS levels [65].

In summary, analysis of the effects of GCs on PG/GAG expression and content in various normal tissues and cells reveals a general trend of inhibition of total PG and GAG biosynthesis, as well as tissue-specific deregulation of the expression of various PG core proteins and a structure of their GAG chains.

## 4. Effects of GCs on PG Expression and GAG Content in Malignant Tissues and Cells

As the key molecular effectors of the cell surface and pericellular space in tissues, PGs regulate the tumor microenvironment (TME) and modulate cancer progression, invasion, and metastasis [75]. Both HSPGs [76,77] and CSPGs [78] play important roles in tumor growth and invasion by driving multiple oncogenic pathways in tumor cells and promoting crucial interactions in the TME. Because GCs are actively used in the treatment of malignant neoplasms, their effects on these key tissue ECM components can be of great functional importance; however, the data on this matter in the literature are very scarce.

DXM is a commonly antiemetic drug in breast cancer therapy. It was shown that DXM time- and dose-dependently increased (up to fourfold) the mRNA and membrane-bound form of syndecan-1 in MCF-7 breast cancer cells, with the greatest effect at a concentration of 10^−6^ M DXM 72 h after treatment, whereas the concentration of the soluble form did not change. Changes in syndecan-1 expression are associated with attenuated biosynthesis of *Osteoprotegerin* and osteoclast formation, identifying tumor-derived syndecan-1 as a novel positive regulator of osteoclastogenesis and a new player in the tumor–bone dialog [79]. DXM at a dose of 0.25 µM was also shown to induce biosynthesis of HS and HA in subconfluent A549 cells by 5–10-fold 72 h after DXM administration [80]. Moreover, DEX administration at a dose of 100 nmol/L significantly decreased expression of CSPG CD44 in GR-positive bladder cancer cells both in mRNA (by 69.7%, *p* < 0.05) and protein levels 48 h after the treatment, although GR silencing in these cells had the opposite effects [81]. Furthermore, treatment of human ovarian cancer cells HO-8910 and SKOV-3 with DXM at a dose of 10^−7^ mol/L did not affect the CD44 protein level or HA secretion during the 6–48 h period after the treatment [82].

The data summarizing the effects of GCs on PGs/GAGs in malignant cells are presented in Table 2.

According to these few data, in tumor cells, GCs tend to increase PG/GAG content (especially HS), unlike in normal cells and tissues, where GCs decrease PG expression and GAG content.

## 5. Effects of GCs on PG Expression and GAG Content in Normal and Malignant Brain Tissues and Cells

GCs are in active use as a companion drug during radiochemotherapy of different brain neoplasms to prevent brain edema, as well as for other malignant tumors. As systemic drugs, GCs affect both the target tumor and the normal tissues, and their long-term use is associated with delayed negative side effects at the molecular, cellular, and clinical level [20,21,22], which also fully applies to normal brain tissue during both corticosteroid therapy and GBM treatment. 

Recent controversial results provided by different laboratories have challenged the widely accepted dogma concerning DXM therapy for GBM [84]. High-dose DXM administration during the initial 3 postoperative weeks resulted in reduced survival, steroid dependency, and infection rate in GBM patients [85], suggesting a need for the use of the lowest dose of GCs for the shortest period to achieve the treatment goals and prevent of steroid-associated complications [19]. Along with the optimization of GC use, it is necessary to investigate more deeply the molecular mechanisms of the effects of GCs and their potential molecular targets in normal brain tissue, including both cellular and extracellular brain components. A significant amount of research data has been obtained to date on the molecular mechanisms of the effects of GCs on various cells of normal brain tissue [3,86,87,88,89] and glioma [90,91], whereas their effect on the brain ECM and PG/GAG content has been much less studied.

### 5.1. Brain ECM

The ECM makes up 20% of brain tissue [92] and takes part in both normal brain physiology and GBM development and progression [93,94,95,96,97], representing an attractive target for the development of new treatment drugs or strategies [98,99,100,101].

In contrast to the matrix of all other tissues, the brain ECM consists mainly of glycosylated molecules such as complex protein–carbohydrate molecules of PGs and carbohydrate molecules of GAGs, which are the most functionally active components within the brain ECM involved in normal and pathological brain functions [102,103,104,105,106]. These macromolecules respond to the various components of conventional anti-GBM treatment, such as chemotherapy with temozolomide (TMZ) [60,61,107] and radiotherapy [108,109]. However, their susceptibility to the effects of GCs, which are commonly used as a concomitant therapy to prevent the risk of cerebral edema, remains largely unexplored.

### 5.2. Effects of GCs on Normal Brain PGs and GAGs

The least-studied issue is the effect of GCs on glycosylated brain tissue, of which PGs and GAGs are the main components. Adding a keyword “brain” to these searches led to significantly fewer articles published on this topic: 44 items for “glucocorticoid brain glycosaminoglycan” among which most are devoted to the use of HA or heparin in the design of modern vehicles for the delivery of drugs or the treatment of various diseases [110,111,112,113]. 

The effects of GCs on brain PGs are mentioned in 19 articles, with 8 for CSPGs and 4 for HSPGs (Figure 1). In vivo administration of therapeutic GC methylprednisolone to female Long–Evans rats (30 mg/kg body weight intravenously immediately after surgery) in the acute spinal cord injury model reduced the expression of *Neurocan* by two to threefold 24 h following treatment (*p* < 0.05) [64]. In AMPA + cyclothiazide-induced reactivated primary cultured astrocytes obtained from Sprague–Dawley rats, pretreatment with methylprednisolone at concentrations of 10 and 50 μM significantly downregulated the expression of *Neurocan* (1.5-fold) and *Phosphacan* (2-fold) (*p* < 0.05). It was shown that the changes in *Neurocan* expression were mediated by the glucocorticoid receptor (GR) [64]. Long-term treatment of adult mice with DXM (5 mg/kg once daily for 6 consecutive weeks) decreased the expression of the basement membrane HSPG *Agrin* by 1.2-fold (*p* < 0.05) in the anterior cortex of mice [62]. Local delivery of DXM in a nitrocellulose-based coating (100 μg DXM in 20 μL nitrocellulose) to rat brains reduced CS content (2.5-fold, *p* < 0.05) one week post implantation [63]. In rat organotypic hippocampal cultures, DXM (0.01–0.5 μM) treatments increased the expression levels of glypican-1 and *Versican* core proteins (5-fold and 10-fold, respectively), whereas 50–200 μM treatments suppressed syndecan-1 and *Biglycan* expression (5-fold and 3-fold, respectively). DXM injection into adult Wistar rats (single injection of 2.5 or 5 mg/kg) in vivo increased overall transcriptional activity of the PGs in the hippocampus in a dose-dependent manner, mainly due to changes in syndecan-1 (+4-fold), glypican-1 (+3-fold), brevican (+7-fold), CSPG4/NG2 (+2-fold), *Decorin* (−2-fold), and *Lumican* (+3-fold) expression 24 h following treatment. In the cortex, only 5 mg/kg of DXM significantly increased the transcriptional activity of the genes. The content of *Decorin* core protein was decreased in the hippocampus and cortex (5-fold, *p* < 0.001 and 4.5-fold, *p* < 0.001, respectively) after 24 h of exposure following DXM (2.5 mg/kg) treatment. In addition, DXM increased CS content in the hippocampus (2-fold, *p* < 0. 001) and decreased CS content in the cortex (2–2.5-fold, *p* < 0. 005), whereas the HS content was decreased in both brain compartments (2–2.5-fold, *p* < 0. 005) [60].

The combination of DXM (2.5 mg/kg on days 1 and 4 of the experiment) with TMZ (30 mg/kg per day for 5 days) resulted in the most profound deterioration in PG composition and content in rat brain tissue at both the core protein and GAG levels. Repetitive low DXM doses (1 mg/kg) were suggested as a more sparing treatment in terms of PG expression compared with high a DXM dose(s), which should be avoided when possible, especially in combination with TMZ [60].

Similar effects of DXM were shown on PG expression in normal mouse brain tissue, where DXM at a dose of 1 mg/kg increased expression of glypican-1 (3.5-fold), syndecan-1 (4.3-fold), and *Versican* (3.1-fold) and decreased CS content (−2.1-fold) in the subcortex, whereas changes in only *Biglycan* expression (+2.7-fold) were detected in the cortex. These DXM-induced changes in PG expression in normal brain tissue were functionally significant, as demonstrated in the GBM relapse animal model. It was shown that ex vivo pretreatment of rat brain organotypic slices with DXM resulted in the accelerated adhesion of U87 GBM cells to the slices, and pretreatment of SCID mice with DXM resulted in a more active in vivo growth of experimental xenograft U87 GBM tumors in the brain of DXM-pretreated SCID mice [61].

These are the main available data on the effects of GCs on brain PGs and GAGs, which represent the first observations of the noticeable effect of GCs on the composition and structure of glycosylated components of the normal brain ECM. 

### 5.3. Effects of Glucocorticoids on PGs and GAGs in Gliomas

Surprisingly, the effect of GCs on the composition and structure of PGs/GAGs in tumor cells and tissues has not yet been practically studied. Although we used various combinations of keywords, including “glucocorticoid”, “proteoglycan”, “glycosaminoglycan”, “heparan sulfate”, “chondroitin sulfate”, “glioma”, “glioblastoma”, and main individual PGs (*Syndecan*, *Glypican*, *Perlecan*, *Aggrecan*, *Neurocan*, *Brevican*, *Decorin*, *Biglycan*, etc.), the PubMed database search, in most cases shows a complete absence of publications. Few known studies in this research field were conducted during the 1980s and 1990s, after which point work in this scientific direction practically ceased. In the only publication retrieved from the database, Mackie et al. showed that treatment of the primary cultures of human gliomas (mainly anaplastic astrocytomas) with 25 μM DXM or 25 μM methylprednisolone affected both cell-surface and serum-released GAGs, although in different ways. DXM slightly increased total GAG content both in the cell medium and pronase digest of the cells, but methylprednisolone decreased the parameter in the culture medium, with an increase in the protease cell digest. Among the three different GAG fractions (HA, HS, and CS/DS), the greatest GC-induced decrease was found in HA [83].

## 6. Conclusions

We present here the available data on the effect of GCs on glycosylated components of various normal and pathological cells and tissues, with a special focus on brain tissue. The presented results indicate a common tendency of GCs to inhibit the expression of the content and/or structure of different PGs and GAGs (HS and CS) in most of the studied tissues and cells in a tissue-specific manner. For some tissues, it is possible to identify the PGs that react most strongly to GCs, whereas in brain tissue, complex changes occur in the expression of various PGs, as well as the content of HS and CS. GC-induced changes in structure and composition of PGs/GAGs lead to the reorganization of the ECM and changes in the tissue structure and functional activity, affecting both tissue development and malignant transformation of cells. 

The possibility of GCs affecting glycosylated components of the ECM might represent a novel mechanism of negative side effects of GC, and further research in this direction might reveal perspective molecular targets to monitor for useful and undesirable effects during GC therapy, as well as its optimization. 

However, the relative scarcity of available data reflects an evident gap in this research field and the necessity of further efforts to investigate the involvement of PGs/GAGs in the molecular mechanism of GC action, as well as negative side effects during systemic GC therapy.

## Figures and Tables

**Table 1:** Effects of glucocorticoids on PG expression and GAG content in normal tissues and cells. Arrows correspond to upregulation (↗) or downregulation (↘) of the PG core protein expression or total PG/GAG content; (=)—no change in the studied parameters; DXM—dexamethasone.

GC	Dose, Regimen	Tissue/Cell/Animal	Detection	Changes in PGs and GAGs	Ref.
**Cartilage and Chondrocytes**					
DXM	2.5 mg/kg for five cycles (5 consecutive days with breaks of 16 days)	Wistar rats	6 months	Total GAGs (↗); sulfated GAGs in TMZ-treated animals (↘)	[39]
	125 μg/kg from gestation day 20 until parturition	Pregnant spiny mice	Offspring	Total PGs in articular and growth cartilages in the offspring (↘)	[44]
	0.2., 0.8, and 1.2 mg/kg once a day from day 12 to day 17 of gestation	Pregnant C57BL/6 mice	Offspring	Gene and protein expression of aggrecan in offspring (↘)	[45]
	1 mg/kg	Rats		Prevented TNFα-induced degradation of aggrecan	[38]
	100 nM and higher	Bovine and human cartilage explants	6 days	Prevented TNFα-induced GAG and PG loss	[36]
	100 nM	Human osteochondral explants	21 days	Prevented a loss of sulfated GAGs from injury- and cytokine-treated explants	[48]
	10^−6^, 10^−7^, and 10^−8^ M	Chondrocytes isolated from mouse knee joints	24 h	Total PG content (↘)	[42]
	1 µM	Primary bovine articular chondrocytes Chondrogenically differentiated mesenchymal stem cells	Day 21, day 14, and days 21–35	GAG production (↗)	[46]
	50–150 μM	Human chondrocytes from knee joints	1–7 days	Aggrecan (↘)	[43]
DXM triamcinolone prednisolone	10^−10^ to 10^−4^ M	Primary equine articular chondrocytes from foals, as well as 7-year-old and adult horses	24 h	*Biglycan* (=) *Decorin* (=)	[47]
Prednisolone	0.5–1 mg/kg/day for 6 months and 13 months	Gottingen miniature pigs	6 months 13 months	No change; GAG content (↘)	[33]
Methylprednisolone acetate	80 mg/mL, 4 injections	Suffolk cross sheep	20 weeks	PG content (↘)	[35]
	0.05 and 0.5 mg/mL	Articular cartilage from1–10-year-old horses		Total PGs (↘)	[41]
Betamethasone		Californian rabbits	1–5 weeks 6–8 weeks	No change; total PG content (↘)	[34]
Triamcinolone acetonide	1.25, 2.5, and 5 mg/mL	Canine cartilage explant		PG content (↘)	[40]
	0.11 mg/mL	Primary canine normal chondrocytes Osteoarthritic chondrocytes		Aggrecan (↘) Aggrecan (↗)	[40]
Cortisone		Rats	1 and 4 weeks	Restored osteoarthritis-induced decrease in total GAG content	[37]
**Lung and Fibroblasts**					
DXM	20 nM	C57Bl/6J mice	24 h	*Betaglycan* at mRNA and protein levels (↗)	[49]
DXM, methylprednisolone, budesonide, and fluticasone		Mouse fibroblasts		*Betaglycan* (↗)	[49]
Cortisol	1.5–4 mg/day	Fetal sheep lung	9 days	Chondrotin-6-sulfate (↘); *Versican* (=); chondroitin-4-sulfate (=)	[50]
Betamethasone	11.4 mg	Fetal sheep lung	24 h and 36 h	*Versican* (↘); chondrotin-6-sulfate (↘); chondroitin-4-sulfate (↘)	[50]
Budesonide	10^−8^ M	Human lung fibroblasts	24 h	Serum-induced total PG increase (↘); *Decorin* (↘); *Versican* (↘)	[51]
**Dermal Fibroblasts**					
DXM	1µM	Human skin fibroblasts		*Decorin* at mRNA and protein levels (↗); *Biglycan* (=); the length of CS/DS chains attached to *Decorin* and *Biglycan* core proteins (=); prevented TGFβ-induced downregulation of *Decorin* and upregulation of *Biglycan*	[52]
	1 µM	Human skin fibroblasts MG-63 osteoblast-like osteosarcoma cell line	1–2 h	Hyaluronan synthase 2 (HAS2) (↘)	[53]
Fluocinolone acetonide, budesonide, and hydrocortisone	10^−11^–10^−5^ M	Human skin fibroblasts		Accumulation of hyaluronic acid (HA) (↘); DS content (↘) in the culture medium on the cell surface and in the cells; decrease in HS content (↘) in culture medium but not on cell surface; considerably less effect	[54]
**Osteoblasts and Bone Marrow Stromal Cells**					
DXM	10^−10^–10^−7^ M	Human osteoblasts (HOBs) and adult human bone marrow stromal cells (BMSC)	7 days	*Decorin* at mRNA and protein levels (↗); *Biglycan* at mRNA and protein levels (↘) in both the conditioned medium and the cell layer	[55]
	10^−10^–10^−7^ M	Osteoblast-like and preosteoblast-like cell lines (murine MC3T3-E1 and rat RCT1)	9–48 h	*Betaglycan* (↗)	[56]
	10^−10^–10^−7^ M	Immature, non-transformed mouse bone-marrow-derived mast cells (mBMMCs)	9–48 h	Serglycin (↗)	[56]
	0.2 mg/kg once a day from gestational day 9 to 20	Rats		Total PG content (↘) in the growth plate	[57]
	100 nM	Young bovine bone marrow stromal cells (BMSCs) seeded on agarose hydrogels		PG biosynthesis (↗)	[58]
	100 nM	Young bovine bone marrow stromal cells (BMSCs) seeded on peptide scaffolds		PG accumulation (↘)	[58]
Hydrocortisone	1 μM	D2XRII, Bl6, and 14F1 bone marrow stromal cell lines		HSPG synthesis (↘) in all three cell lines; altered pattern of CS/DSPGs in the culture media by DS1 and DS3 (↗) DS2 (↘)	[59]
**Brain**					
DXM	1 mg/kg 2.5 and 5 mg/kg	Wistar rats	24 h	PG expression (=); overall transcriptional activity of PGs (↗); syndecan-1 (↗); glypican-1 (↗); brevican (↗); CSPG4/NG2 (↗); *Lumican* (↗); *Decorin* (↘); CS content in hippocampus (↗); CS content in cortex (↘); HS content (↘) in both	[60]
	1 mg/kg	SCID mice		Glypican-1 (↗); syndecan-1 (↗); *Versican* (↗); CS content (↘) in the subcortex; *Biglycan* in cortex (↗)	[61]
	5 mg/kg once daily for 6 consecutive weeks	Adult mice		*Agrin* (↘)	[62]
	100 μg DEX in 20 μL nitrocellulose Local delivery into rat brain	Rats	7 days	CS content (↘)	[63]
	0.01–0.5 μM 50–200 μM	Rat organotypic hippocampal cultures		Glypican-1 (↗); *Versican* (↗); syndecan-1 (↘); *Biglycan* (↘)	[60]
Methylprednisolone	30 mg/kg body weight intravenously immediately after SCI surgery	Female Long–Evans rats (acute spinal cord injury model)	24 h	*Neurocan* (↘)	[64]
	Pretreatment 10 and 50 μM	AMPA + cyclothiazide-induced reactivated primary cultured astrocytes (Sprague–Dawley rats)		*Neurocan* (↘) *Phosphacan* (↘)	[64]
**Other Tissue and Cells**					
DXM	16 mg	Patients undergoing colorectal, pancreas, or liver surgery	Postoperative day 1	Plasma syndecan-1 level (=); heparan sulfate content (↘)	[65]
	0.55 μM	Human eyes in ex vivo perfusion organ culture	7 days and 14–21 days after treatment	No change In total GAG (CS, DS, HS, and HA) content in human trabecular meshwork (↗)	[66]
		Rat mesangial cells (RMCs) and human mesangial cells (HMCs)		Total CS/DSPGs synthesis and secretion (↘); *Decorin* (↘) *Biglycan* (↗)	[67]
DXM, hydrocortisone Aldosterone	200 or 400 ng/mL 200 ng/mL 500 ng/mL	Hepatic stellate cells (HSCs)		*Betaglycan* mRNA level (↗)	[68]
DXM triamcinolone	1 μM 1 μM	Primary cultures of human tenocytes from explants of healthy human patellar tendon		Total PG content (↘)	[69]
Methylprednisolone		New Zealand Black/White F1 mice	Onset of murine lupus nephritis (5 months of age)	Expression, distribution, and intensity of HSPG staining in renal cortex (↘)	[70]
	2 mg/kg single preoperative dose	Patients undergoing gastrointestinal surgery	1 and 3 days after surgery	Syndecan-1 (↘); HS content (↘) in endothelial glycocalyx	[71]

**Table 2:** Effects of glucocorticoids on PG expression and GAG content in malignant tissues and cells. Arrows correspond to upregulation (↗) or downregulation (↘) of the PG core protein expression or total PG/GAG content, (=)—no change in the studied parameters. DXM—dexamethasone.

GC	Dose	Tissue/Cells/Animals	Detection	Changes in PGs and GAGs	Ref.
DXM	Up to 10^−6^ M	MCF-7 breast cancer cells	72 h	mRNA and membrane-bound form of syndecan-1 (↗); concentration of the soluble form of syndecan-1 (=)	[79]
	0.25 µM	Subconfluent A549 cells	72 h	Heparan sulfate (↗); hyaluronic acid (↗)	[80]
	100 nmol/L	Bladder cancer cells	48 h	CD44 expression (↘)	[81]
	10^−7^ mol/L	HO-8910 and SKOV-3 human ovarian cancer cells	6–48 h	CD44 protein level (=); hyaluronic acid secretion (=)	[82]
DXM methylprednisolone	25 μM	Primary cultures of human gliomas (mainly anaplastic astrocytomas)		Total GAG content (↗); both in the cell medium and pronase digest of the cells’ total GAG content: in the culture medium (↘); in the protease cell digest (↗)	[83]

## Data Availability

Not applicable.
